# Semaglutide 2.4 mg in Participants With Metabolic Dysfunction‐Associated Steatohepatitis: Baseline Characteristics and Design of the Phase 3 ESSENCE Trial

**DOI:** 10.1111/apt.18331

**Published:** 2024-10-16

**Authors:** Philip N. Newsome, Arun J. Sanyal, Kristiane A. Engebretsen, Iris Kliers, Laura Østergaard, Denise Vanni, Elisabetta Bugianesi, Mary E. Rinella, Michael Roden, Vlad Ratziu

**Affiliations:** ^1^ Roger Williams Institute of Liver Studies, Faculty of Life Sciences and Medicine King's College London and King's College Hospital London UK; ^2^ College of Medical and Dental Sciences University of Birmingham Birmingham UK; ^3^ VCU School of Medicine Stravitz‐Sanyal Institute for Liver Disease and Metabolic Health Richmond Virginia USA; ^4^ Novo Nordisk A/S Copenhagen Denmark; ^5^ Department of Medical Sciences University of Turin Turin Italy; ^6^ Division of Gastroenterology, Hepatology and Nutrition University of Chicago Chicago Illinois USA; ^7^ Department of Endocrinology and Diabetology, Medical Faculty and University Hospital Düsseldorf Heinrich Heine University Düsseldorf Düsseldorf Germany; ^8^ Institute for Clinical Diabetology, German Diabetes Center Leibniz Center for Diabetes Research at Heinrich Heine University Düsseldorf Düsseldorf Germany; ^9^ German Center for Diabetes Research, Partner Düsseldorf München‐Neuherberg Germany; ^10^ Sorbonne Université, Institute for Cardiometabolism and Nutrition Hospital Pitié‐Salpêtrière, INSERM UMRS 1138 CRC Paris France

**Keywords:** ESSENCE, MASH, MASLD, semaglutide

## Abstract

**Background:**

Semaglutide, a glucagon‐like peptide‐1 receptor agonist, has demonstrated potential beneficial effects in metabolic dysfunction‐associated steatohepatitis (MASH).

**Aims:**

To describe the trial design and baseline characteristics of the ‘Effect of Semaglutide in Subjects with Non‐cirrhotic Non‐alcoholic Steatohepatitis’ (ESSENCE) trial (NCT04822181).

**Methods:**

ESSENCE is a two‐part, phase 3, randomised, multicentre trial evaluating the effect of subcutaneous semaglutide 2.4 mg in participants with biopsy‐proven MASH and fibrosis stage 2 or 3. The primary objective of Part 1 is to demonstrate that semaglutide improves liver histology compared with placebo. The two primary endpoints are: resolution of steatohepatitis and no worsening of liver fibrosis, and improvement in liver fibrosis and no worsening of steatohepatitis. The Part 2 objective is based on clinical outcomes. The current work reports baseline characteristics of the first 800 randomised participants which includes demographics, laboratory parameters, liver histology, non‐invasive tests and presence of metabolic dysfunction‐associated steatotic liver disease (MASLD) cardiometabolic criteria.

**Results:**

Of 800 participants, 250 (31.3%) had fibrosis stage 2 and 550 (68.8%) had fibrosis stage 3. In the overall population, mean (standard deviation [SD]) age was 56 (11.6) years, 57.1% were female, mean (SD) body mass index was 34.6 (7.2) kg/m^2^, 55.5% had type 2 diabetes and > 99% had at least one MASLD cardiometabolic criterion according to the published definition.

**Conclusion:**

The ESSENCE baseline population includes participants with clinically significant fibrosis stages 2 and 3. Although MASLD cardiometabolic criteria were not a requirement for study enrolment, almost all participants (> 99%) had at least one MASLD cardiometabolic criterion.

**Trial Registration:**

NCT04822181

## Graphical abstract

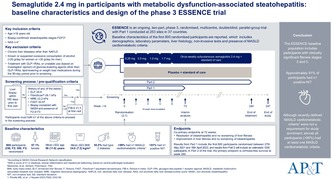

## 1 Introduction

Metabolic dysfunction‐associated steatohepatitis (MASH; formerly known as non‐alcohol‐related steatohepatitis [NASH]) is a potentially severe form of metabolic dysfunction‐associated steatotic liver disease (MASLD; formerly non‐alcoholic fatty liver disease [NAFLD]), characterised by chronic inflammation that drives progressive fibrosis which may lead to cirrhosis and/or hepatocellular carcinoma [1, 2]. MASLD is estimated to affect approximately 30% of adults worldwide, [3, 4] with an estimated prevalence of MASH in people with MASLD of around 20%. Obesity, insulin resistance, metabolic alterations (e.g., dysglycaemia and dyslipidaemia) and systemic low‐grade (or subclinical) inflammation are considered root causes of MASLD [5, 6].

Until recently, there were no approved pharmacological treatments for MASH. However, in March 2024, an oral, liver‐directed, thyroid hormone receptor beta‐selective agonist (resmetirom) received accelerated approval by the US Food and Drug Administration (FDA) for the treatment of MASH with fibrosis stage 2 or 3. Definitive approval will be conditional upon demonstration of clinically meaningful benefit in the ongoing phase 3 trials of resmetirom [7, 8].

Evidence suggests that semaglutide, a glucagon‐like peptide‐1 receptor agonist (GLP‐1RA), may have a beneficial effect in MASH [9, 10, 11]. In a phase 2 trial of 320 participants with biopsy‐confirmed MASH and fibrosis stage 1–3, treatment with once‐daily subcutaneous semaglutide 0.4 mg resulted in a significantly higher proportion of participants achieving MASH resolution with no worsening of fibrosis compared with placebo (59% vs. 17%; *p* < 0.001) [12]. Given that the leading cause of death in people with MASLD is cardiovascular disease, [13] therapeutic interventions for MASLD should, ideally, also improve cardiometabolic risk factors, in addition to improving liver condition [14]. In the phase 2 trial population, treatment with semaglutide was associated with dose‐dependent improvements in cardiometabolic parameters (e.g., weight loss and glycated haemoglobin levels) [12]. Additionally, in another phase 2 trial of 71 participants with MASH and compensated cirrhosis, once‐weekly subcutaneous semaglutide 2.4 mg also achieved improvements in cardiometabolic measures, with no additional safety concerns in the cirrhotic population [15]. These initial findings provide an early indication that semaglutide not only improves liver histology but also cardiometabolic risk factors in patients with MASH. To build on this evidence base, larger trials are required. Here, we report the trial design and baseline characteristics (including demographics, laboratory parameters, histology and non‐invasive test [NIT] results) of the first cohort of 800 participants randomised in the phase 3 ‘Effect of Semaglutide in Subjects with Non‐cirrhotic Non‐alcoholic Steatohepatitis’ (ESSENCE) trial.

## 2 Methods

### 2.1 Trial Design and Endpoints

**FIGURE 1:**
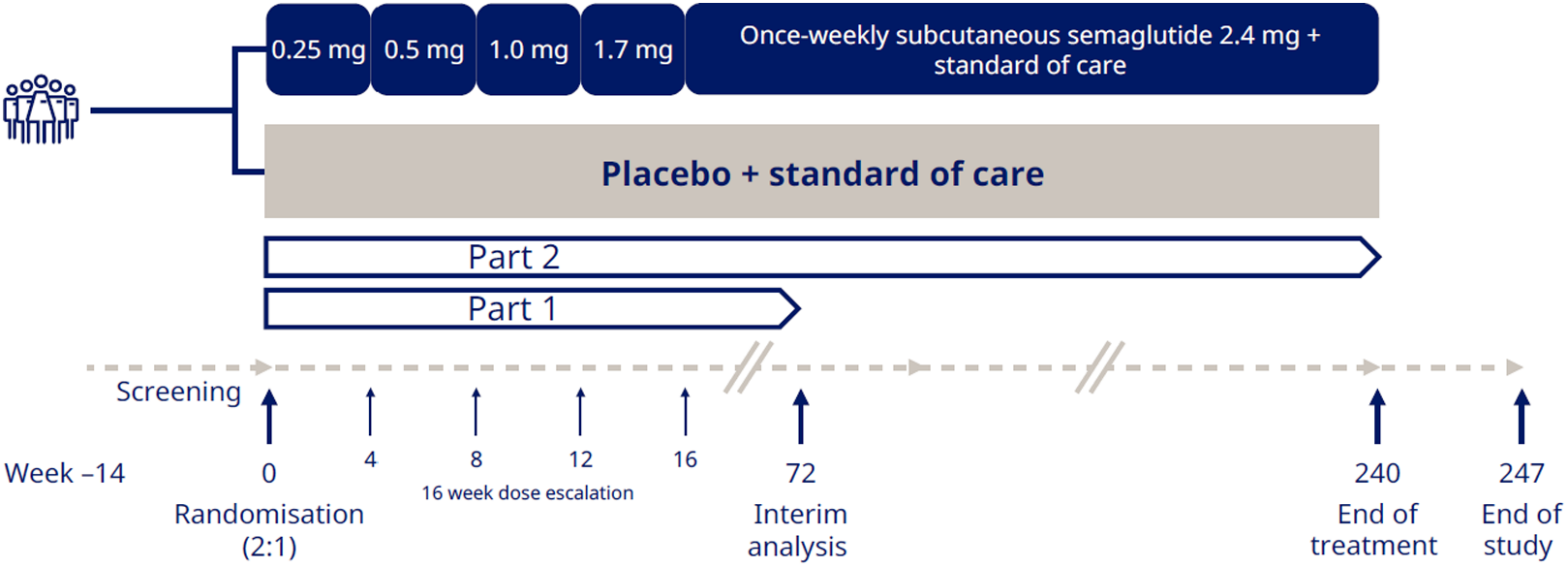
ESSENCE trial design.

ESSENCE (NCT04822181) is an ongoing two‐part, phase 3, randomised, multicentre, double‐blind, parallel‐group trial in participants with MASH and fibrosis stage 2 or 3. The trial is designed to recruit approximately 20% of the total population (estimated *N* = 1200) with fibrosis stage 2. Part 1 was conducted at 253 sites in 37 countries. As part of the screening process, a pre‐qualification approach is employed with the aim of increasing the likelihood of participants having fibrosis stage 2 or 3 and, hence, a decreased histological screen failure rate. Participants are required to fulfil one or more of several pre‐qualification criteria: a historical liver biopsy within 180 days prior to the first screening visit that could be centrally assessed; a history of any of the following NIT results: enhanced liver fibrosis (ELF) ≥ 9.8, liver stiffness ≥ 9.1 kPa (assessed using vibration‐controlled transient elastography [VCTE]/FibroScan), magnetic resonance elastography ≥ 3.2 kPa, FibroScan‐aspartate transaminase (FAST) score ≥ 0.67; biopsy consistent with NASH and presence of fibrosis stage 2 or fibrosis stage 3 (which could occur at any time point outside of the 180 days); or a fibrosis‐4 (FIB‐4) score ≥ 1.3 measured at first visit. During a 14‐week screening period, after fulfilling at least one of the pre‐qualification criteria, there are several central laboratory tests, clinical assessments and in‐trial liver biopsy (if no historical liver biopsy within 180 days of the first screening visit) required to eventually evaluate eligibility of participants for randomisation. The screening period is followed by a 240‐week treatment period (Part 1: 0–72 weeks; Part 2: 0–240 weeks), and a 7‐week follow‐up period (Figure 1). Results from Part 1 include the first 800 participants randomised between 27 May 2021 and 18 April 2023, and results from Part 2 will include an estimated 1200 participants. Participants are randomised 2:1 to receive once‐weekly subcutaneous semaglutide 2.4 mg or placebo, both added to standard of care (investigators were encouraged to optimise treatment for type 2 diabetes [T2D], dyslipidaemia, and cardiovascular risk management according to a standard of care guidance document). Randomisation is stratified based on the presence of T2D at screening, fibrosis stage (2 or 3) and, for regulatory purposes, geographic region (Japan, East Asia excluding Japan or rest of the world). The treatment period includes a 16‐week dose‐escalation phase for subcutaneous semaglutide. During dose escalation, one or more dose steps can be prolonged or the dose lowered if the actual dose is not tolerated. If the designated target dose of once‐weekly subcutaneous semaglutide 2.4 mg is not tolerated, participants may stay at a lower dose level. It is recommended that a participant makes at least one attempt to re‐escalate to the designated target dose as per the investigator's discretion.

### 2.2 Objectives and Endpoints

The primary objective of Part 1 is to demonstrate that treatment with subcutaneous semaglutide 2.4 mg improves liver histology compared with placebo in participants with MASH and fibrosis stage 2 or 3. In Part 1, the two primary endpoints are: (1) resolution of steatohepatitis and no worsening of liver fibrosis and (2) improvement in liver fibrosis and no worsening of steatohepatitis. Resolution of steatohepatitis is defined as an NAFLD activity score (NAS) of 0–1 for inflammation, 0 for ballooning and any value for steatosis (according to the NASH Clinical Research Network [CRN] classification). Fibrosis is graded on the NASH CRN fibrosis scale from 0 to 4, with an improvement in fibrosis defined as ≥ 1 grade improvement. No worsening of steatohepatitis is defined as no increase from baseline in NAS score for ballooning, inflammation or steatosis. Confirmatory secondary endpoints at week 72 are change in body weight, resolution of steatohepatitis and improvement in liver fibrosis, and change in Short Form 36 (SF‐36) Bodily Pain. In Part 2 of the trial, the primary endpoint is cirrhosis‐free survival at week 240, with the aim of demonstrating that treatment with semaglutide 2.4 mg lowers the risk of liver‐related clinical events compared with placebo in subjects with MASH and fibrosis stage 2 or 3. All endpoints are reported in Table S1.

### 2.3 Eligibility

Eligible participants are required to be aged ≥ 18 years with histological presence of steatohepatitis with fibrosis stage 2 or fibrosis stage 3 according to the NASH CRN classification, and a NAS of ≥ 4, with a score of ≥ 1 in steatosis, lobular inflammation and hepatocyte ballooning, all based on a central pathologist evaluation of a baseline liver biopsy. Key exclusion criteria includes participants with documented causes of chronic liver disease other than NAFLD; known or suspected alcohol consumption higher than 20 g/day for women and 30 g/day for men or alcohol dependence as assessed by the alcohol use disorders identification test (AUDIT questionnaire); and presence or a history of ascites, variceal bleeding, hepatic encephalopathy, spontaneous bacterial peritonitis or liver transplantation at randomisation. Participants are also excluded if they test positive for hepatitis B surface antigen (HBsAg), anti‐human immunodeficiency virus or hepatitis C virus (HCV) RNA at screening or any known presence of HCV RNA or HBsAg within 2 years of screening. Treatment‐related exclusion criteria includes vitamin E (at doses greater than or equal to 800 IU/day) or pioglitazone or medications approved for the treatment of MASH (including resmetirom which was conditionally approved during the trial) which has not been at a stable dose for 90 days prior to the screening visit; GLP‐1RAs for 90 days prior to screening; and glucose‐lowering agent(s) (other than GLP‐1RAs), lipid‐lowering medication or weight‐loss medication not stable in the opinion of the investigator for 90 days prior to screening. In addition, for participants with historical liver biopsies taken more than 90 days prior to screening, treatment should be at a stable dose from time of biopsy until screening.

### 2.4 Central Pathologist Evaluation

In the ESSENCE trial, liver histology evaluation is performed by a total of six central pathologists. All the participant's digitalised liver histology slides are independently reviewed by one of the three expert pairs of pathologists. The participant's digitalised liver histology slides are designated randomly to one of the pairs, where each one of the central pathologists separately analyses the same participant's digitalised liver histology slides. All central pathologists are blinded towards each other's evaluations (unless a consensus call is required), each trial participant's ID and treatment arm assignment. The central pathologists within a pair evaluate and agree (or not) on whether the liver biopsy is comparable with steatohepatitis (confirmation of diagnosis only at screening), fibrosis stage according to NASH CRN fibrosis stage categories 0–4 and scoring of NAS components. If there is any discrepancy in these features within the central pathologist pair evaluations of a participant's digitalised liver histology slides, the central pathologists from the referred pair are notified by the central laboratory and instructed to perform a consensus call. If a consensus on evaluation cannot be reached within the referred pair, a third pathologist is asked to independently evaluate the referred feature(s) and this last evaluation is considered final.

### 2.5 Analysis

A pre‐specified statistical testing strategy is used to control for multiplicity across the two primary and confirmatory secondary endpoints (Figure S1). Evidence of efficacy for semaglutide 2.4 mg versus placebo on liver histology is considered established if statistical superiority is demonstrated for at least one of the two primary liver histology endpoints. Additionally, Part 1 of ESSENCE employs a group sequential design allowing two confirmatory efficacy analyses: one at an intermediate stage involving the first 800 randomised participants and another at the final stage involving the planned 1200 randomised participants. Overall, the study has a statistical power of 95% to detect a responder proportion of 20.0% in the semaglutide 2.4 mg arm compared with 10.0% in the placebo arm. In the current baseline analysis, baseline data for the first 800 randomised participants were pooled and assessed by fibrosis stage 2 or 3 and analysed descriptively using mean (standard deviation [SD]) and/or median (interquartile range) for continuous variables, and number and percentage for categorical variables. The presence and quantification of the new MASLD cardiometabolic criteria (defined as body mass index [BMI] ≥ 25 kg/m^2^ [23 kg/m^2^ Asia] or waist circumference > 94 cm [males] or 80 cm [females] or ethnicity‐adjusted equivalent; fasting serum glucose ≥ 5.6 mmol/L [100 mg/dL] or 2‐h post‐load glucose levels ≥ 7.8 mmol/L [≥ 140 mg/dL] or glycated haemoglobin [HbA_1c_] ≥ 5.7% [39 mmol/L] or T2D or treatment for T2D; blood pressure ≥ 130/85 mmHg or specific antihypertensive drug treatment; plasma triglycerides ≥ 1.70 mmol/L [150 mg/dL] or lipid‐lowering treatment; plasma high‐density lipoprotein [HDL] cholesterol ≤ 1.0 [40 mg/dL] [males] and ≤ 1.3 mmol/L [50 mg/dL] [females] or lipid‐lowering treatment) [1] were also analysed in the randomised population. Two‐hour post‐load glucose levels could not be assessed as an oral glucose tolerance test was not included as a laboratory measure in the trial and blood pressure criterion (≥ 130/85 mmHg) was replaced in the current analysis by a medical history of systemic arterial hypertension. The number of MASLD cardiometabolic criteria fulfilled by the 800 randomised participants were stratified by NAS score and also by fibrosis stage. The baseline characteristics of the 800 enrolled participants were compiled after completion of enrolment and without knowledge of the randomisation assignment.

### 2.6 Participants

The trial is conducted in accordance with the principles of the Declaration of Helsinki and International Conference on Harmonisation Good Clinical Practice guidelines. All trial participants provided written informed consent before data collection.

## 3 Results

The data cut‐off date for the current analysis was 10 December 2023. Of the 800 randomised participants, 250 (31.3%) had fibrosis stage 2 and 550 (68.8%) had fibrosis stage 3 (Table 1).

**TABLE 1:** ESSENCE baseline characteristics.

	Fibrosis stage 2 (*n* = 250)	Fibrosis stage 3 (*n* = 550)	Total (*N* = 800)
Age, years, mean ± (SD)	53.1 (12.7)	57.4 (10.8)	56.0 (11.6)
Female	133 (53.2)	324 (58.9)	457 (57.1)
Race			
Asian	64 (25.6)	152 (27.6)	216 (27.0)
White	168 (67.2)	372 (67.6)	540 (67.5)
Black/African American	1 (0.4)	4 (0.7)	5 (0.6)
Other^a^	14 (5.6)	17 (3.1)	31 (3.9)
Missing	3 (1.2)	5 (0.9)	8 (1.0)
Ethnicity			
Not Hispanic/Latino	199 (79.6)	433 (78.7)	632 (79.0)
Hispanic/Latino	45 (18.0)	101 (18.4)	146 (18.3)
Not reported	4 (1.6)	13 (2.4)	17 (2.1)
Missing	2 (0.8)	3 (0.5)	5 (0.6)
Type 2 diabetes^b^	116 (46.4)	328 (59.6)	444 (55.5)
BMI, kg/m^2^, mean ± (SD)	35.3 (7.6)	34.2 (7.0)	34.6 (7.2)
< 25	15 (6.0)	38 (6.9)	53 (6.6)
≥ 25 to < 30	44 (17.6)	121 (22.0)	165 (20.6)
≥ 30 to < 35	80 (32.0)	171 (31.1)	251 (31.4)
≥ 35	111 (44.4)	219 (39.8)	330 (41.3)
Missing	0 (0)	1 (0.2)	1 (0.1)
MASLD cardiometabolic criteria^c^ fulfilled			
0	1 (0.4)	0 (0)	1 (0.1)
1	14 (5.6)	15 (2.7)	29 (3.6)
2	23 (9.2)	46 (8.4)	69 (8.6)
3	50 (20.0)	83 (15.1)	133 (16.6)
4	76 (30.4)	146 (26.5)	222 (27.8)
5	86 (34.4)	260 (47.3)	346 (43.3)

*Note:* Data are number and percentages of participants unless otherwise stated.

Abbreviations: BMI, body mass index; HbA_1c_, glycated haemoglobin; HDL, high‐density lipoprotein; MASLD, metabolic dysfunction‐associated steatotic liver disease; SD, standard deviation; T2D, type 2 diabetes.

^a^ Includes but not limited to Native Hawaiian/other Pacific Islander and American Indian/Alaska Native.

^b^ T2D defined as a medical history of T2D and/or HbA_1c_ ≥ 6.5% (≥ 48 mmol/mol).

^c^ MASLD cardiometabolic criteria defined as BMI ≥ 25 kg/m^2^ [23 kg/m^2^ Asia] or waist circumference > 94 cm (males) or 80 cm (females) or ethnicity‐adjusted equivalent; fasting serum glucose ≥ 5.6 mmol/L (100 mg/dL) or 2‐h post‐load glucose levels ≥ 7.8 mmol/L (≥ 140 mg/dL) or HbA_1c_ ≥ 5.7% (39 mmol/L) or T2D or treatment for T2D; blood pressure ≥ 130/85 mmHg (replaced in the current analysis by medical history of systemic arterial hypertension) or specific antihypertensive drug treatment; plasma triglycerides ≥ 1.70 mmol/L (150 mg/dL) or lipid‐lowering treatment; plasma HDL cholesterol ≤ 1.0 (40 mg/dL) (males) and ≤ 1.3 mmol/L (50 mg/dL) (females) or lipid‐lowering treatment [1].

### 3.1 Baseline Characteristics

In the overall population, mean (SD) age was 56 (11.6) years and 57.1% were female. Regarding race, 67.5% were White, 27.0% Asian and 0.6% Black/African American (4.9% were reported as other or missing). Approximately half of the participants (44.5%) did not have T2D (40.4% of those without T2D had fibrosis stage 3). The mean (SD) BMI was 34.6 (7.2): 27.2% of participants had a BMI < 30 kg/m^2^, of which 6.6% had a BMI < 25 kg/m^2^. Baseline characteristics were generally similar across fibrosis stages 2 and 3, although numbers of females, participants with T2D, BMI categories < 25 and ≥ 25 to < 30 kg/m^2^ and the presence of five MASLD cardiometabolic criteria were numerically higher for fibrosis stage 3 versus fibrosis stage 2.

Cardiometabolic risk factors were highly prevalent in this population. Almost all participants (> 99%) had at least one MASLD cardiometabolic criteria according to published definitions of MASLD, [1] with approximately half (43.3%) meeting all five MASLD cardiometabolic criteria. A numerically greater proportion of participants with fibrosis stage 3 met all five MASLD cardiometabolic criteria versus participants with fibrosis stage 2 (47.3% vs. 34.4%, respectively) (Table S2). Liver aminotransferases were within normal range (central laboratory reference ranges: upper limits of normal of 55 U/L for alanine transaminase and 34 U/L for aspartate transaminase) in 26.3% of participants (fibrosis stage 2, 28.0% vs. fibrosis stage 3, 26.0%). Serum levels of total cholesterol, non‐esterified fatty acids and triglycerides were numerically similar between fibrosis stages (Table 2). Mean (SD) NAS was 5.05 (0.95): 5.11 (0.95) in participants with fibrosis stage 3 and 4.92 (0.93) for fibrosis stage 2 (Table 3).

**TABLE 2:** ESSENCE baseline laboratory parameters.

	Fibrosis stage 2 (*n* = 250)	Fibrosis stage 3 (*n* = 550)	Total (*N* = 800)
Alanine aminotransferase (U/L)^a^	72.0 (47.5) [13.0–309.0]	66.2 (40.8) [11.0–299.0]	68.1 (43.1) [11.0–309.0]
Aspartate aminotransferase (U/L)^b^	51.9 (30.4) [14.0–202.0]	53.5 (29.9) [13.0–322.0]	53.0 (30.0) [13.0–322.0]
Gamma‐glutamyl transferase (U/L)	78.1 (95.7)	90.7 (86.9)	86.7 (89.9)
Alkaline phosphatase (U/L)	86.2 (27.9)	89.0 (31.4)	88.1 (30.4)
Total bilirubin (mg/dL)	0.68 (0.34)	0.66 (0.30)	0.66 (0.31)
Albumin (g/dL)	4.4 (0.3)	4.3 (0.3)	4.3 (0.3)
INR, ratio	1.1 (0.1)	1.1 (0.2)	1.1 (0.1)
Thrombocytes, 10^9^/L	245.1 (66.0)	223.6 (64.9)	230.4 (66.0)
Total cholesterol (mmol/L), median (IQR)	4.79 (4.18–5.51)	4.62 (3.99–5.33)	4.69 (4.09–5.40)
HDL cholesterol (mmol/L), median (IQR)	1.16 (0.98–1.34)	1.15 (0.98–1.39)	1.15 (0.98–1.37)
LDL cholesterol (mmol/L), median (IQR)	2.79 (2.19–3.53)	2.62 (2.04–3.27)	2.68 (2.10–3.35)
Triglycerides (mmol/L), median (IQR)	1.68 (1.26–2.16)	1.65 (1.26–2.20)	1.66 (1.26–2.18)
Non‐esterified fatty acids (mmol/L), median (IQR)	0.56 (0.40–0.76)	0.60 (0.43–0.77)	0.59 (0.42–0.76)
High‐sensitivity C‐reactive protein (mg/L)	5.93 (6.24)	5.22 (5.86)	5.44 (5.98)
HbA_1c_ (%)	6.4 (1.0)	6.6 (1.1)	6.5 (1.1)

*Note:* Data are mean ± (SD) [range; minimum–maximum] unless otherwise stated.

Abbreviations: HbA_1c_, glycated haemoglobin; HDL, high‐density lipoprotein; INR, international normalised ratio; IQR, interquartile range; LDL, low‐density lipoprotein; SD, standard deviation.

^a^ Upper limit of normal, 55 U/L.

^b^ Upper limit of normal, 34 U/L.

**TABLE 3:** ESSENCE baseline histology (NAS).

	Fibrosis stage 2 (*n* = 250)	Fibrosis stage 3 (*n* = 550)	Total (*N* = 800)
Steatosis score			
0	0 (0)	0 (0)	0 (0)
1	69 (27.6)	221 (40.2)	290 (36.3)
2	127 (50.8)	237 (43.1)	364 (45.5)
3	54 (21.6)	92 (16.7)	146 (18.3)
Hepatocyte ballooning score			
0	0 (0)	0 (0)	0 (0)
1	122 (48.8)	198 (36.0)	320 (40.0)
2	128 (51.2)	352 (64.0)	480 (60.0)
Lobular inflammation score			
0	0 (0)	0 (0)	0 (0)
1	135 (54.0)	186 (33.8)	321 (40.1)
2	114 (45.6)	343 (62.4)	457 (57.1)
3	1 (0.4)	21 (3.8)	22 (2.8)
NAS, mean ± (SD)	4.92 (0.93)	5.11 (0.95)	5.05 (0.95)

*Note:* Data are number and percentages of participants unless otherwise stated.

Abbreviations: NAS, non‐alcoholic fatty liver disease activity score; SD, standard deviation.

In the total population, 34.5% of participants had FIB‐4 < 1.3 (45.2% and 29.6% with fibrosis stage 2 and 3, respectively). Mean (SD) FIB‐4 score increased with severity of fibrosis stage (1.52 [0.93] for fibrosis stage 2 vs. 1.91 [1.00] for fibrosis stage 3, with scores ranging from 0.28–7.04 and 0.33–7.43, respectively). Mean (SD) ELF score was 10.0 (1.0): 43.5% of participants had an ELF score < 9.8. The proportion of participants with a score of < 9.8 was 62.4% for fibrosis stage 2 and 34.9% for those with fibrosis stage 3. ELF score values ranged from 7.6–12.4 in participants with fibrosis stage 2 to 7.4–13.2 in participants with fibrosis stage 3. Mean (SD) VCTE/FibroScan controlled attenuation parameter (CAP) value was 329 (46) dB/m: 335 (44) dB/m for fibrosis stage 2 and 327 (47) dB/m for fibrosis stage 3. Mean (SD) VCTE/FibroScan liver stiffness value was 12.8 (6.9) kPa (10.4 [5.3] kPa in participants with fibrosis stage 2 and 13.9 [7.3] for fibrosis stage 3). Liver stiffness values of < 8 kPa were observed in 15.3% of participants (26.8% and 10.0% with fibrosis stage 2 vs. fibrosis stage 3, respectively). Approximately 91% of the trial population had at least one positive NIT based on FIB‐4 ≥ 1.3, VCTE ≥ 8.1 or ELF ≥ 9.8 (Table 4).

**TABLE 4:** ESSENCE baseline NITs.

	Fibrosis stage 2 (*n* = 250)	Fibrosis stage 3 (*n* = 550)	Total (*N* = 800)
**FIB‐4**, median (IQR) [range; min–max]	1.36 (0.91–1.87) [0.28–7.04]	1.68 (1.19–2.38) [0.33–7.43]	1.56 (1.11–2.27) [0.28–7.43]
< 1.0	74 (29.6)	77 (14.0)	151 (18.9)
≥ 1.0 to < 1.30	39 (15.6)	86 (15.6)	125 (15.6)
≥ 1.30 to ≤ 2.67	110 (44.0)	279 (50.7)	389 (48.6)
> 2.67	24 (9.6)	93 (16.9)	117 (14.6)
Missing	3 (1.2)	15 (2.7)	18 (2.3)
ELF score, mean ± (SD) [range; min–max]	9.5 (0.8) [7.6–12.4]	10.2 (0.9) [7.4–13.2]	10.0 (1.0) [7.4–13.2]
CAP VCTE (dB/m), mean ± (SD)	335 (44)	327 (47)	329 (46)
Liver stiffness VCTE (kPa), mean ± (SD)	10.4 (5.3)	13.9 (7.3)	12.8 (6.9)
FIB‐4 ≥ 1.3, VCTE ≥ 8.1 or ELF ≥ 9.8			
0 NITs fulfilled^a^	39 (15.6)	32 (5.8)	71 (8.9)
1 NIT fulfilled	102 (40.8)	122 (22.2)	224 (28.0)
2 NITs fulfilled	71 (28.4)	212 (38.5)	283 (35.4)
3 NITs fulfilled	38 (15.2)	184 (33.5)	222 (27.8)

*Note:* Data are number and percentages of participants unless otherwise stated.

Abbreviations: CAP, controlled attenuation parameter; ELF, enhanced liver fibrosis; FIB‐4, fibrosis‐4; IQR, interquartile range; max, maximum; min, minimum; NIT, non‐invasive test; SD, standard deviation; VCTE, vibration‐controlled transient elastography.

^a^ Likely attributable to differences between cut‐offs, that is, VCTE ≥ 9.1 used in the trial but VCTE ≥ 8.1 reported here to align with guidelines.

## 4 Discussion

We present comprehensive baseline data for the first 800 participants randomised in Part 1 of the ESSENCE trial. ESSENCE has two parts with distinctive objectives and endpoints. In Part 1, the aim of the trial is to demonstrate that treatment with subcutaneous semaglutide 2.4 mg improves liver histology compared with placebo in participants with MASH and fibrosis stage 2 or 3. The primary histological endpoints at week 72 were explored previously in a phase 2b trial [12] and align with FDA guidelines for the development of treatments for MASH [7]. To best evaluate these endpoints, it is crucial that the trial population reflects as closely as possible the population to which the intervention will be targeted.

By definition, MASLD is associated with cardiometabolic risk factors [16] and MASH is associated with a greater cardiovascular risk than MASLD [17, 18]. Although the presence of cardiometabolic risk factors was not a requirement for ESSENCE trial enrolment, almost all (> 99%) of the participants met at least one MASLD cardiometabolic criterion according to the published definition, [1] with greater numbers of cardiometabolic criteria seen in those with higher NAS and higher fibrosis stage. Interestingly, the proportion of participants with up to four MASLD cardiometabolic criteria was similar within fibrosis stage 2 and stage 3 subgroups. However, a ‘turning point’ was the higher proportion of participants with five MASLD cardiometabolic criteria in the fibrosis stage 3 subgroup. These data clearly highlight the cardiometabolic burden of MASH in the ESSENCE trial population. In a recently reported population‐based cohort study of > 230,000 patients with T2D, the rate of major adverse liver outcomes increased progressively with increasing numbers of metabolic syndrome traits (hypertension, obesity, hypertriglyceridaemia, a low level of HDL and albuminuria) [19]. This observation suggests a strong association between poor metabolic health and liver disease risk. Taken together, we consider the ESSENCE cohort to be highly relevant to the trial objectives.

The enrolment of participants into MASH clinical trials is challenging with 65%–87% of screened participants not meeting eligibility criteria [20, 21, 22]. Population‐based screening for MASH is not currently recommended; [23] however, clinical practice guidelines are generally aligned on target populations for screening of individuals at risk of fibrosis and disease progression, in particular, those with T2D, obesity and elevated transaminases [23, 24, 25]. Although cardiometabolic comorbidities are highly prevalent in the ESSENCE cohort, nearly half of participants did not have T2D. Over one quarter did not have obesity (6.6% had a BMI < 25 kg/m^2^ suggestive of a subgroup of lean MASH) and over one quarter had normal liver enzymes. These findings show that using simple clinical factors alone would have resulted in missing clinically significant disease. Clinical practice guidelines universally recommend FIB‐4 as a first‐line NIT for fibrosis risk stratification, [23, 24, 25] with FIB‐4 < 1.3 corresponding to a low risk of advanced fibrosis. However, a recent study reported that a substantial number of participants were misclassified by FIB‐4 as low risk despite being at high risk for advanced fibrosis based on liver stiffness measurement by vibration‐controlled transient elastography, when guidelines for fibrosis risk identification were applied to a general population of National Health and Nutrition Examination Survey participants [26]. Moreover, although the negative predictive value of the FIB‐4 score is high, approximately 10% of individuals with advanced fibrosis will be missed [27]. In the current work, the proportion that would have been missed is as high as one third of randomised participants. Therefore, it is important to recognise that FIB‐4 has greater utility in low prevalence populations such as primary care as opposed to liver clinics where it can miss patients due to the higher prevalence of liver fibrosis. Despite the implementation of pre‐qualification criteria in the ESSENCE trial (where participants had to meet at least one of the criteria), randomised participants had a diverse range of values for several NITs (those with no positive NITs were enrolled based on past biopsy). This highlights the importance of evaluating multiple NITs since if only one were to be used, our findings might imply that participants with clinically relevant disease could be missed. It should be noted that ESSENCE is not designed or powered to validate NITs, and that FIB‐4 score validation was originally performed in a cohort of high‐risk participants, not participants with MASLD [28].

A key strength of the ESSENCE trial, discussed above, is that its cohort satisfies the new MASLD diagnostic criteria [1]. As a result, the relationship between the MASLD diagnostic criteria, fibrosis stage and NAS histological components can be assessed. It is acknowledged that there is a limitation in the ESSENCE trial concerning the inclusion of underrepresented racial and ethnic populations within the first 800 participants randomised. This is demonstrated by a scarce number of some ethnic/race groups compared with the most prevalent groups represented in the trial. Part 1 of ESSENCE is an interim analysis performed after 72 weeks of treatment and does not evaluate liver‐related clinical outcomes. Longer‐term data from Part 2 are required to evaluate the effect of semaglutide on liver‐related clinical outcomes such as hepatic decompensation or liver transplantation and all‐cause mortality.

## 5 Conclusions

The ESSENCE baseline population includes participants with clinically significant fibrosis stages 2 and 3. Although MASLD cardiometabolic criteria were not a requirement for trial enrolment, almost all participants (≥ 99%) had at least one MASLD cardiometabolic criterion, emphasising the metabolic burden of MASH. Approximately 91% of the trial population had at least one positive NIT consistent with clinically significant fibrosis. Baseline data suggest that many participants with clinically significant disease could be missed using currently recommended criteria for first‐line screening in clinical trials of MASH.

## Author Contributions

**Philip N. Newsome:** writing – review and editing. **Arun J. Sanyal:** writing – review and editing. **Kristiane A. Engebretsen:** conceptualization, methodology, investigation, project administration, writing – review and editing. **Iris Kliers:** conceptualization, investigation, methodology, writing – review and editing, project administration. **Laura Østergaard:** conceptualization, methodology, data curation, investigation, formal analysis, project administration. **Denise Vanni:** conceptualization, methodology, investigation, project administration, writing – review and editing. **Elisabetta Bugianesi:** writing – review and editing. **Mary E. Rinella:** writing – review and editing. **Michael Roden:** writing – review and editing. **Vlad Ratziu:** writing – review and editing.

## Conflicts of Interest

Philip N. Newsome reports grants from Novo Nordisk, and has received consulting fees from Boehringer Ingelheim, Madrigal and Novo Nordisk. Philip N. Newsome also reports honoraria as a speaker from AiCME, Echosens and Novo Nordisk; support for attending meetings for Novo Nordisk; and participation on an advisory board for Boehringer Ingelheim, GSK, Madrigal, Novo Nordisk and Sagimet. Elisabetta Bugianesi served as a consultant or advisory board member for Boehringer Ingelheim, Gilead, Intercept, Merck, Novo Nordisk, Pfizer and ProSciento, and as a speaker for Gilead, Intercept, Merck, Novo Nordisk and Pfizer. Elisabetta Bugianesi has also received a research grant from Gilead for fatty liver research. Vlad Ratziu received consulting fees from Boehringer Ingelheim, GSK, Madrigal, Novo Nordisk, ProSciento and Sagimet, and research grants (to institution) from MSD. Mary E. Rinella consults for 89bio, Akero, Boehringer Ingelheim, CytoDyn, GSK, HistoIndex, Intercept, Madrigal, NGM Bio, Novo Nordisk, Sagimet and Sonic Incytes. Mary E. Rinella has received fees for consulting or participation in advisory boards for Boehringer Ingelheim, Echosens, Eli Lilly and Novo Nordisk. Mary E. Rinella also reports honoraria as a speaker for CME events sponsored by Boehringer Ingelheim, Madrigal and Novo Nordisk. Michael Roden received lecture fees or served on advisory boards for AstraZeneca, Echosens, Eli Lilly, Madrigal, Merck‐MSD, Novo Nordisk and Target RWE, and performed investigator‐initiated research with support from Boehringer Ingelheim, Novo Nordisk and Nutricia/Danone to the German Diabetes Center (DDZ). Arun J. Sanyal consults for and advises AstraZeneca and Avant Santé. Arun J. Sanyal also consults for and has received grants from Akero, BMS, Eli Lilly, Intercept, Madrigal and Novo Nordisk. Arun J. Sanyal consults for and owns stock in Rivus, and also consults for 89bio, AGED Diagnostics, Albireo, Alnylam, Altimmune, Boehringer Ingelheim, Echosens, Genentech, Gilead, GSK, HistoIndex, Mallinckrodt, Merck, NGM Bio, Novartis, PathAI, Pfizer, Poxel, Regeneron, Salix, Siemens, Surrozen, Takeda, Terns and Zydus. Arun J. Sanyal owns stock in Durect, Exalenz, Genfit, Indalo, Inversago and Tiziana, and has received royalties from Elsevier and Wolters Kluwer. Kristiane A. Engebretsen, Iris Kliers, Laura Østergaard, Denise Vanni are employees and stockholders of Novo Nordisk A/S.

## Authorship

*Guarantor of the article*: Philip N. Newsome. All authors approved the final version of the manuscript.

## Supporting information


Data S1.

## Data Availability

Data will be shared with bona fide researchers who submit a research proposal approved by the independent review board. The data will be available after research completion and approval of product and product use in both the EU and USA. The end date depends on external retention requirements. Individual participant data will be shared in data sets in a de‐identified and anonymised format. Information about data access request proposals can be found at novonordisk‐trials.com.
