# Supplementary material for: Semaglutide 2.4 mg in Participants With Metabolic Dysfunction‐Associated Steatohepatitis: Baseline Characteristics and Design of the Phase 3 ESSENCE Trial
Source: Aliment Pharmacol Ther. 2024 Oct 16;60(11-12):1525–33. doi: 10.1111/apt.18331 (PMC11599791; doi:10.1111/apt.18331)
Supplement: Supplementary file 1 — Data S1. [file APT-60-1525-s001.docx]

**Supplementary Material**

Supplement to: Newsome PN, Sanyal AJ, et al. Semaglutide 2.4 mg in participants with metabolic dysfunction-associated steatohepatitis: baseline characteristics and design of the phase 3 ESSENCE trial

**TABLE S1** ESSENCE Part 1 endpoints.

| **Primary outcome measures** |
| --- |
| Resolution of steatohepatitis and no worsening of liver fibrosis |
| Improvement in liver fibrosis and no worsening of steatohepatitis |
| **Secondary outcome measures** |
| Change in body weight |
| Resolution of steatohepatitis and improvement in liver fibrosis |
| Change in SF-36 Bodily Pain |
| **Secondary outcome measures – histology** |
| Improvement in steatohepatitis with at least a two-point reduction in NAS and no worsening of fibrosis |
| Change in histology-assessed liver collagen proportionate area |
| Worsening in steatohepatitis |
| Improvement in histology-assessed ballooning |
| Improvement in histology-assessed inflammation |
| Improvement in histology-assessed steatosis |
| NASH resolution (ballooning of 0, inflammation of 0–1) and ≥2-point NAS reduction with no worsening of fibrosis |
| Progression of liver fibrosis in patients with fibrosis stage 2 at baseline |
| **Secondary outcome measures – imaging** |
| Change in CAP values assessed by transient elastography (FibroScan^®^) |
| Changes in liver stiffness values assessed by transient elastography (FibroScan^®^) |
| **Secondary outcome measures – NITs** |
| Change in ELF score |
| Change in FAST |
| Change in Pro-C3 |
| **Secondary outcome measures – laboratory assessments** |
| Change in ALT |
| Change in AST |
| Change in inflammation assessed by hsCRP |
| Change in HbA1c |
| Change in triglyceride |
| Change in free fatty acids |
| Change in LDL cholesterol |
| Change in HDL cholesterol |
| **Secondary outcome measures – PROs** |
| Changes in SF-36 (v2.0 acute) Physical Component Summary |
| Changes in SF-36 Mental Component Summary |
| Changes in NASH-CHECK Abdominal Pain |

Abbreviations: ALT, alanine transaminase; AST, aspartate transaminase; CAP, controlled attenuation parameter; ELF, enhanced liver fibrosis; FAST, FibroScan^®^-AST; HbA1c, glycated haemoglobin; HDL, high-density lipoprotein; hsCRP, high sensitivity C-reactive protein; LDL, low-density lipoprotein; NAS, non-alcoholic fatty liver disease activity score; NASH, non-alcohol-related steatohepatitis; NIT, non-invasive test; PRO, patient-reported outcome; Pro-C3, pro-peptide of type III collagen; SF-36, Short Form 36.

**TABLE S2** Number of MASLD cardiometabolic criteria fulfilled by NAS in the overall population and by fibrosis stage.

|  |  |  | **NAS score** |  |  |
| --- | --- | --- | --- | --- | --- |
|  | 4  *n* (%) | 5  *n* (%) | 6  *n* (%) | ≥7  *n* (%) | Total  *N* (%) |
| **Number** | 267 | 299 | 168 | 66 | 800 |
| **MASLD criteria* fulfilled** | |  |  |  |  |
| 0 | 1 (0.4) | 0 (0.0) | 0 (0.0) | 0 (0.0) | 1 (0.1) |
| 1 | 13 (4.9) | 13 (4.3) | 2 (1.2) | 1 (1.5) | 29 (3.6) |
| 2 | 18 (6.7) | 35 (11.7) | 13 (7.7) | 3 (4.5) | 69 (8.6) |
| 3 | 58 (21.7) | 32 (10.7) | 31 (18.5) | 12 (18.2) | 133 (16.6) |
| 4 | 65 (24.3) | 84 (28.1) | 53 (31.5) | 20 (30.3) | 222 (27.8) |
| 5 | 112 (41.9) | 135 (45.2) | 69 (41.1) | 30 (45.5) | 346 (43.3) |
| **Fibrosis stage 2** |  |  |  |  |  |
| **Number** | 102 | 85 | 45 | 18 | 250 |
| **MASLD criteria* fulfilled** | |  |  |  |  |
| 0 | 1 (1.0) | 0 (0.0) | 0 (0.0) | 0 (0.0) | 1 (0.4) |
| 1 | 6 (5.9) | 5 (5.9) | 2 (4.4) | 1 (5.6) | 14 (5.6) |
| 2 | 6 (5.9) | 12 (14.1) | 5 (11.1) | 0 (0.0) | 23 (9.2) |
| 3 | 30 (29.4) | 9 (10.6) | 7 (15.6) | 4 (22.2) | 50 (20.0) |
| 4 | 25 (24.5) | 27 (31.8) | 16 (35.6) | 8 (44.4) | 76 (30.4) |
| 5 | 34 (33.3) | 32 (37.6) | 15 (33.3) | 5 (27.8) | 86 (34.4) |
| **Fibrosis stage 3** |  |  |  |  |  |
| **Number** | 165 | 214 | 123 | 48 | 550 |
| **MASLD criteria* fulfilled** | |  |  |  |  |
| 0 | 0 (0.0) | 0 (0.0) | 0 (0.0) | 0 (0.0) | 0 (0.0) |
| 1 | 7 (4.2) | 8 (3.7) | 0 (0.0) | 0 (0.0) | 15 (2.7) |
| 2 | 12 (7.3) | 23 (10.7) | 8 (6.5) | 3 (6.8) | 46 (8.4) |
| 3 | 28 (17.0) | 23 (10.7) | 24 (19.5) | 8 (16.7) | 83 (15.1) |
| 4 | 40 (24.2) | 57 (26.6) | 37 (30.1) | 12 (25.0) | 146 (26.5) |
| 5 | 78 (47.3) | 103 (48.1) | 54 (43.9) | 25 (52.1) | 260 (47.3) |

Abbreviations: BMI, body mass index; HbA_1c_, glycated haemoglobin; HDL, high-density lipoprotein; MASLD, metabolic dysfunction-associated steatotic liver disease; NAS, non-alcoholic fatty liver disease activity score; T2D, type 2 diabetes.

**^*^**MASLD cardiometabolic criteria defined as BMI ≥25 kg/m^2^ [23 kg/m^2^ Asia] or waist circumference >94 cm (males) 80 cm (females) or ethnicity adjusted equivalent; fasting serum glucose ≥5.6 mmol/L (100 mg/dL) or 2-hour post-load glucose levels ≥7.8 mmol/L (≥140 mg/dL) or HbA_1c_ ≥5.7% (39 mmol/L) or T2D or treatment for T2D; blood pressure ≥130/85 mmHg (replaced in the current analysis by medical history of systemic arterial hypertension) or specific antihypertensive drug treatment; plasma triglycerides ≥1.70 mmol/L (150 mg/dL) or lipid-lowering treatment; plasma HDL cholesterol ≤1.0 (40 mg/dL) (males) and ≤1.3 mmol/L (50 mg/dL) (females) or lipid‑lowering treatment (Rinella ME, Lazarus JV, Ratziu V, Francque SM, Sanyal AJ, Kanwal F, et al. A multisociety Delphi consensus statement on new fatty liver disease nomenclature. J Hepatol. 2023;79(6):1542-56)

**FIGURE S1** Testing procedure.


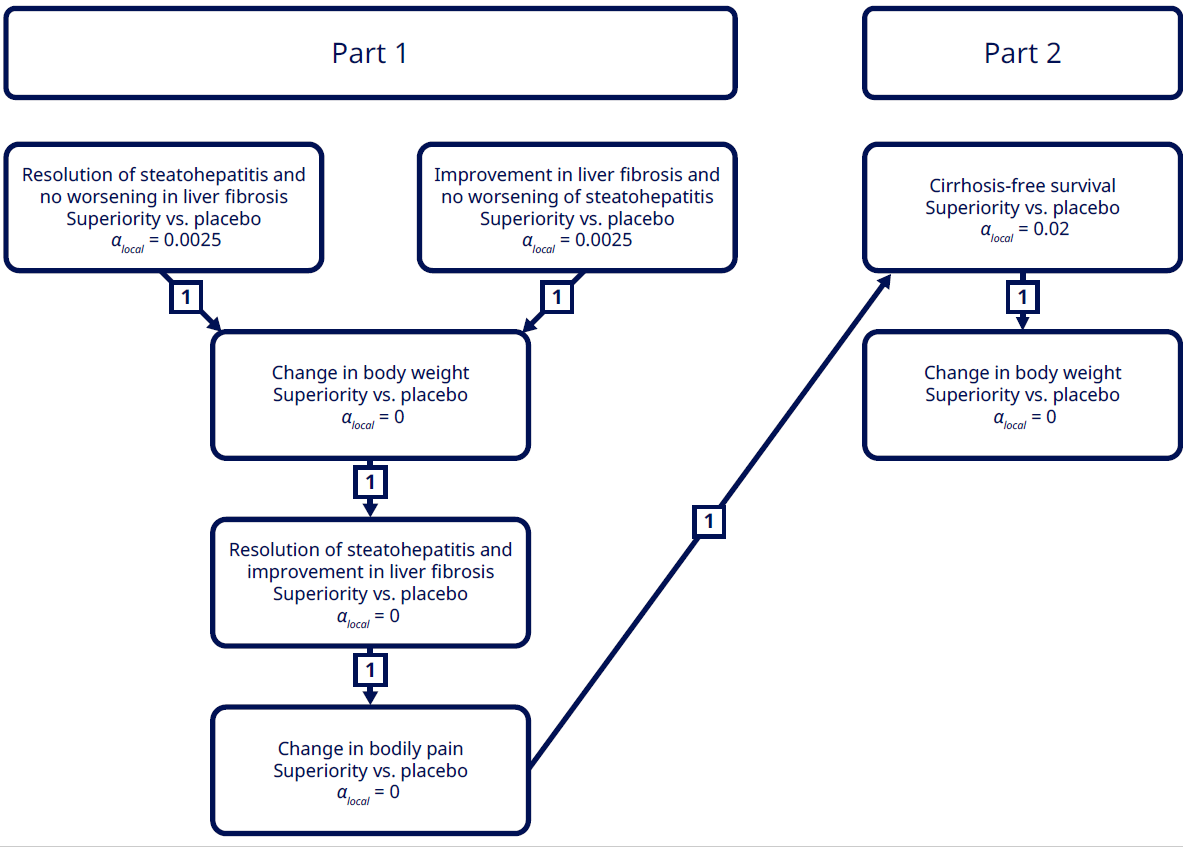


Graphical representation of the testing procedure as described by Bretz and colleagues (Bretz F, Maurer W, Brannath W, Posch M. A graphical approach to sequentially rejective multiple test procedures. Stat Med 2009;28:586-604). α*_local_* is the pre-allocated level of alpha for a given endpoint. The arrows represent which tests the alpha is sent to after a test is rejected. The numbers in the boxes on the arrows represent the fraction of alpha being passed on. In case the hypothesis is not rejected no alpha is passed on.
The overall significance level of α (0.025) (for one-sided testing) is initially split between the two parts of the trial. A local significance level α*_local_* (0.005) is allocated to Part 1, and α*_local_* 0.02 is allocated to Part 2. The α*_local_* for Part 1 is further split into α*_local_* 0.0025 allocated to the test on resolution of steatohepatitis and no worsening in liver fibrosis, and α*_local_* 0.0025 allocated to the test on improvement in liver fibrosis and no worsening in steatohepatitis. If both tests confirm superiority, then α*_local_* 0.005 will be reallocated to the test on change in body weight from baseline to 72 weeks. If only one of the tests confirms superiority, then α*_local_* 0.0025 will be reallocated to the test on change in body weight. If none of the tests confirm superiority, the test on change in body weight will not be performed.
